# Investigation of Fusion between Nanosized Lipid Vesicles and a Lipid Monolayer Toward Formation of Giant Lipid Vesicles with Various Kinds of Biomolecules

**DOI:** 10.3390/mi12020133

**Published:** 2021-01-26

**Authors:** Koki Kamiya, Chika Arisaka, Masato Suzuki

**Affiliations:** 1 Division of Molecular Science, Graduate School of Science and Technology, Gunma University 1-5-1 Tenjin-cho, Kiryu, Gunma 376-8515, Japan; t201a006@gunma-u.ac.jp; 2 Department of Chemistry and Biochemistry, Faculty of Science and Technology, Gunma University 1-5-1 Tenjin-cho, Kiryu, Gunma 376-8515, Japan; t170a072@gunma-u.ac.jp

**Keywords:** artificial cell membrane, giant lipid vesicles, vesicle fusion, asymmetric lipid bilayer, microfluidic device

## Abstract

We determined the properties of fusion between large unilamellar vesicles (LUVs) and the lipid monolayer by measuring the fluorescence intensity of rhodamine-conjugated phospholipids in cell-sized lipid vesicles. The charge of LUVs (containing cationic lipids) and lipid droplets (containing anionic lipids) promoted lipid membrane fusion. We also investigated the formation of cell-sized lipid vesicles with asymmetric lipid distribution using this fusion method. Moreover, cell-sized asymmetric ganglioside vesicles can be generated from the planar lipid bilayer formed at the interface between the lipid droplets with/without LUVs containing ganglioside. The flip-flop dynamics of ganglioside were observed on the asymmetric ganglioside vesicles. This fusion method can be used to form asymmetric lipid vesicles with poor solubility in *n*-decane or lipid vesicles containing various types of membrane proteins for the development of complex artificial cell models.

## 1. Introduction

Artificial cell membranes, including lipid vesicles or planar lipid bilayers consisting of a phospholipid bilayer, are used for investigating the dynamics of lipid membranes and the functions of membrane proteins, and they are applied in drug delivery systems [1,2,3,4,5]. Planar bilayer lipid membrane (BLM) systems, using a droplet contact method, have been developed to form membranes between droplet/droplet interfaces, referred to as droplet interface bilayers (DIBs) [6,7]. This DIB system is very useful for measuring the ion currents of nanopores and ion channels [8,9,10], creating tissue models [11], and generating giant lipid vesicles by applying a pulsed jet flow against the planar lipid bilayer [12,13]. In particular, DIBs facilitate the functional analyses of membrane proteins, the multi-step reactions between lipid droplets, and the development of artificial cell models mimicking living cells such as asymmetric lipid bilayers [14,15,16,17]. For instance, when membrane proteins such as ion channels are reconstituted into the planar lipid bilayer for measuring the ion currents of ion channels, the membrane protein-reconstituted large unilamellar vesicle (LUV) of 100–200 nm in diameter is fused with the lipid monolayer in the lipid droplet [9,18,19]. The LUV fusion with the lipid monolayer aids in reconstituting phospholipids, which differ from the lipid monolayer. Although the fusion between LUVs and the lipid monolayer is important for integrating the membrane proteins or phospholipids into the planar lipid bilayer, the properties of the fusion between LUVs and the lipid monolayer, which relies on the LUV incubation time, associated lipid components, and lipid monolayers, are not clear. Therefore, to control the reconstitution amount of biomolecules (membrane proteins and lipids) on the planar lipid bilayer for analysis, determining the fusion properties between LUVs and the lipid monolayer by changing the fusion periods and the lipid components is necessary.

**Figure 1:**
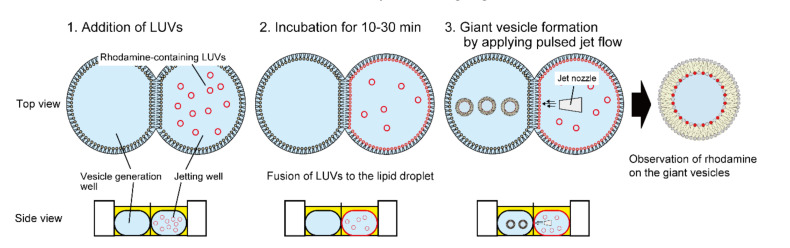
Schematic images of a fusion system between large unilamellar vesicles (LUVs) and lipid droplets. (**1**) Lipid droplets (water-in-oil emulsion) containing LUVs with rhodamine-conjugated lipids were formed in a double-well device. (**2**) LUVs were fused to lipid droplets. (**3**) After incubation for 10–30 min at 23–25 °C, cell-sized lipid vesicles were generated by applying a pulsed jet flow against the planar lipid bilayer at the interface between the lipid droplets. Rhodamine fluorescence of the cell-sized lipid vesicles was observed under a confocal laser scanning microscope (CLSM).

In this study, we determine the properties of the fusion between LUVs and lipid monolayers using a water-in-oil (w/o) emulsion as the planar lipid bilayer by changing the charge of the lipid compositions and the incubation time for fusion between LUVs and the lipid monolayer. The fusion of LUVs to the lipid monolayer is observed as schematically described in Figure 1. In brief, lipid droplets containing rhodamine-conjugated LUVs are incubated to allow for the formation of a planar lipid bilayer at the interface between the lipid droplets containing LUVs and those without LUVs, and giant lipid vesicles are then generated by applying a pulsed jet flow against the planar lipid bilayer [17,20,21]. The fusion properties of LUVs with the lipid monolayer are estimated according to the fluorescence intensities of rhodamine on the giant lipid vesicles observed by confocal laser scanning microscopy (CLSM). First, an optimal concentration of LUVs is determined; second, the fusion of LUVs to lipid droplets is investigated by changing the lipid charges (negatively and positively charged phospholipids or zwitterionic phospholipids) of the LUVs and lipid droplets. Lastly, to verify the usefulness of this LUV fusion property, the formation of giant lipid vesicles with asymmetric lipid distribution (rhodamine-conjugated lipids or ganglioside) is examined. The flip-flop dynamics of ganglioside are also observed on the cell-sized asymmetric ganglioside vesicles.

## 2. Materials and Methods

### 2.1. Materials

1,2-Dioleoyl-*sn*-glycero-3-phosphocholine (DOPC), 1,2-dioleoyl-*sn*-glycero-3-phospho-l-serine (sodium salt) (DOPS), 1,2-dioleoyl-3-trimethylammonium-propane (methyl sulfate salt) (DOTAP), ganglioside GM1 (bovine brain), and 1,2-dioleoyl-*sn*-glycero-3 phosphoethanolamine– *N*-(lissamine rhodamine B sulfonyl) (ammonium salt) (Rh-DOPE) were purchased from Avanti Polar Lipids, Inc. (Alabaster, AL, USA). Cholera toxin subunit B (recombinant) Alexa Fluor 488 conjugate was purchased from Thermo Fisher Scientific (Waltham, MA, USA). *n*-Decane was purchased from Sigma Aldrich (St. Louis, MO, USA). 

### 2.2. Device Fabrication 

A double-well device was fabricated by assembling a poly(methyl methacrylate) (PMMA) plate (Acylight; Mitsubishi Rayon) using an automated computer-aided design and computer-aided manufacturing (CAD/CAM) modeling machine (MM-100; Modia Systems Co., Inc., Koshigaya, Japan). The double-well device was formed with two overlapping cylindrical wells (4, 3, and 3 mm in diameter, depth, and overlap width, respectively) [17]. A separator with one aperture of 200 or 500 µm in diameter was prepared from 75 µm of PMMA film. The separator was assembled between the wells. The bottom of this double well was attached to a 1 mm thick acrylic substrate by thermocompression bonding. A microjet nozzle with a 20–40 µm inner diameter orifice was fabricated from a thin-walled glass capillary with a 1 mm outer diameter using a micropipette puller (PC-10; Narishige Group, Tokyo, Japan). The nozzle tip was curved to 30° using a microforge (MF-900; Narishige Group, Tokyo, Japan) (Figure S1a, Supplementary Materials). The microjet nozzle was connected to a liquid dispenser system (SJVC3000; Sansei Tech, Japan) (Figure S1b, Supplementary Materials).

### 2.3. Formation of Large Unilamellar Vesicles (LUVs)

LUVs composed of 2 mM DOPC containing 76 nM Rh-DOPE, 2 mM DOPC/DOPS (molar ratio 1:1) containing 76 nM Rh-DOPE, 2 mM DOPC/DOTAP (molar ratio 9:1) containing 76 nM Rh-DOPE, and 2 mM DOPC/DOPS/GM1 (molar ratio 4:5:1) containing 76 nM Rh-DOPE were prepared via the extruder method. The phospholipids of these compartments in chloroform were evaporated into a glass microtube under flowing argon gas. Films of the phospholipids were formed at the bottom of the tube. The films were hydrated by a buffer solution (10 mM HEPES/140 mM NaCl pH 7.4) and vortex-mixed with 2 mM lipid vesicle solutions. The lipid vesicle solutions were filtered using an Avanti mini-extruder with polycarbonate membranes of 0.1 µm pore diameter. The LUV diameters were measured using a dynamic scattering method (ELSZ-2000; Otsuka Electronics, Osaka, Japan).

### 2.4. Fusion of LUVs to a Lipid Droplet

Briefly, 6 µL of 50 mM DOPC solution dissolved in *n*-decane was added to each well. Next, 22 µL of 10 mM HEPES/140 mM NaCl (pH 7.4) buffer containing 500 mM sucrose and 0.5, 0.25, and 0.125 mM DOPC LUVs was added to the well of the jetting side, and 23 µL of 10 mM HEPES/140 mM NaCl (pH 7.4) buffer containing 500 mM glucose was added to the well of the vesicle generation side. After incubation for 30 min at 23–25 °C, the pulsed jet flow was applied to the planar lipid bilayer using the liquid dispenser system (application time: 3–4 ms, applications pressure: 0.3 MPa). Next, 8 µL of solution containing vesicles was collected from the well of the vesicle generation side. To observe the giant lipid vesicles in the chamber, those containing sucrose buffer which sank in the glucose buffer due to differences in density between the outside and inside of giant lipid vesicles were observed using a confocal laser scanning microscope (CLSM) (FV-1200; Olympus, Tokyo, Japan).

### 2.5. Fusion of LUVs to a Lipid Droplet at Different Incubation Times and Different Lipid Comportments

The lipid droplets were composed of DOPC or DOPC/DOPS (molar ratio 1:1). The LUVs were composed of DOPC, DOPC/DOPS (molar ratio 1:1), and DOPC/DOTAP (molar ratio 9:1). The incubation times for fusion of 0.5 mM (final concentration) LUVs to the lipid droplet were 10, 20, or 30 min. The fusion experimental procedure is described below. Briefly, 6 µL of 50 mM lipid solution dissolved in *n*-decane was added to each well. Next, 22 µL of 10 mM HEPES/140 mM NaCl (pH 7.4) buffer containing 500 mM sucrose and 0.5 mM LUVs was added to the well of the jetting side, and 23 µL of 10 mM HEPES/140 mM NaCl (pH 7.4) buffer containing 500 mM glucose was added to the well of the vesicle generation side. After incubation for 10, 20, or 30 min at 23–25 °C, pulsed jet flow was applied to the planar lipid bilayer using a liquid dispenser system (application time: 3–4 ms, applications pressure: 0.3 MPa). Next, 8 µL of solution containing vesicles was collected from the well of the vesicle generation side. The solution was observed using a CLSM (FV-1200; Olympus).

### 2.6. Fusion between LUVs and Giant Lipid Vesicles at Different Incubation Times and Lipid Comportments

Initially, 1 mM giant lipid vesicles were prepared via the gentle hydration method. Lipid films (DOPC/DOPS (molar ratio 95:5) and DOPC/DOPS (molar ratio 1:1)) were hydrated with a buffer solution (10 mM HEPES/140 mM NaCl (pH 7.4)) and incubated for 4 h at 23 °C, and the giant lipid vesicles were formed. Next, 10 µL of 1 mM LUVs (DOPC, DOPC/DOPS (molar ratio 1:1), and DOPC/DOTAP (molar ratio 9:1)) were added to 10 µL of 1 mM giant lipid vesicle solution. The solution was incubated for 10, 20, or 30 min at 23–25 °C. The solution was observed using a CLSM.

### 2.7. CLSM Observation of the Giant Lipid Vesicles

Rhodamine-DOPE of lipid vesicles was observed using a CLSM (FV-1200; Olympus) with an oil-immersion lens (60×) and a diode laser (559 nm) for rhodamine and a diode laser (473 nm) for Alexa Fluor 488. The fluorescence intensities of rhodamine and Alexa Fluor 488 on the vesicles were measured using the ImageJ software.

### 2.8. Formation of Asymmetric GM1 Vesicles

To form asymmetric GM1-conatining giant lipid vesicles, 0.5 mM (final concentration) GM1 LUVs (DOPC/DOPS/GM1 (molar ratio 4:5:1) containing 76 nM Rh-DOPE) were added to the lipid droplet of the jetting well or the vesicle formation well. After incubation for 20 min at 23–25 °C, asymmetric GM1 vesicles were generated by applying a pulsed jet flow against the planar lipid bilayer. Next, 1 µL of Alexa Fluor 488-conjugated cholera toxin b (ctxB) (fourfold diluted) was added to 49 µL of the outer solution of the asymmetric lipid vesicles. After incubation for 20 min at 23–25 °C, Alexa Fluor 488-conjugated ctxB on the asymmetric GM1 vesicles was observed using the CLSM.

## 3. Results and Discussion

### 3.1. Determination of Optimal LUV Concentrations for DIB

**Figure 2:**
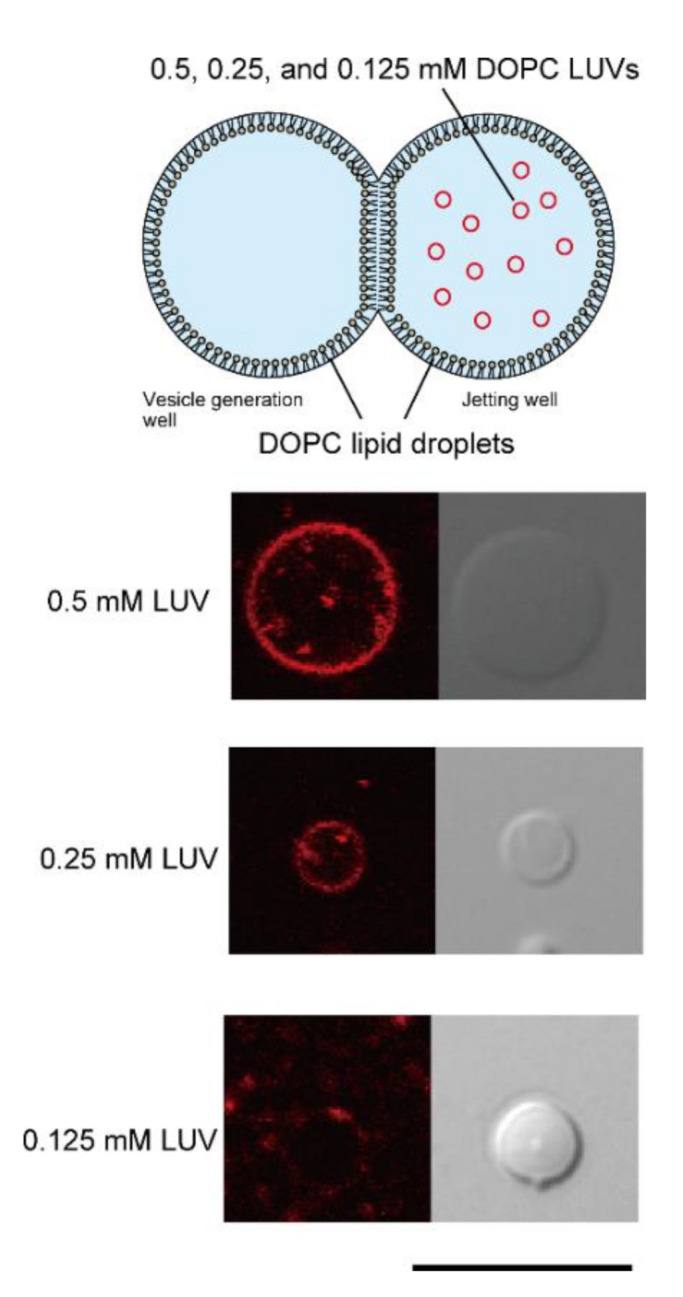
Determination of the optimal LUV concentration for fusion to lipid droplets. First, 1,2-dioleoyl-*sn*-glycero-3-phosphocholine (DOPC) lipid droplets containing 0.5, 0.25, and 0.125 mM (final concentration) DOPC LUVs containing rhodamine-1,2-dioleoyl-*sn*-glycero-3 phosphoethanolamine (DOPE) were prepared in the jetting well, and giant lipid vesicles were generated by the pulsed jet flow method. Typical microscopic images of giant lipid vesicles containing 0.5, 0.25, and 0.125 mM (final concentration) DOPC LUVs in the DOPC lipid droplets of the jetting well are shown. Scale bar, 10 µm.

First, to determine the optimal LUV concentration for fusion with lipid bilayer droplets in the double-well device, final concentrations of 0.5, 0.25, and 0.125 mM DOPC LUVs containing rhodamine-DOPE were added to DOPC lipid droplets in the jet flow well (Figure 2). The diameter of DOPC LUVs was 117.9 nm. After a 30 min incubation, using the pulsed jet flow method, giant lipid vesicles were formed from the planar lipid bilayer, which was formed via the contact between lipid droplets. The fusion of the LUVs to the lipid droplets was confirmed by rhodamine fluorescence on the giant lipid vesicles. When 0.125 mM DOPC LUVs were applied to DOPC lipid droplets, rhodamine fluorescence was detected in 20.8% (5/24) of giant lipid vesicles. When the low concentration of LUVs was added to the lipid droplet in the double-well device, the absolute amount of rhodamine-lipids on the lipid monolayer was low. Therefore, the amount of rhodamine-lipids of giant lipid vesicles generated under this condition was below the measurement limit of our microscope system. In contrast, when 0.5 mM DOPC LUVs were added, 88.2% (15/17) of giant lipid vesicles showed rhodamine fluorescence. Therefore, we selected 0.5 mM LUVs for fusion with lipid droplets into the double-well device. 

The stabilities of the planar lipid bilayer were confirmed by assessing the retention of DOPC LUVs containing rhodamine-DOPE into the lipid droplet chamber. When a final concentration of 0.5 mM DOPC LUVs was added to one side of the chamber of the DOPC droplet, no movement of the LUV was observed during incubation for 60 min (Figure S2, Supplementary Materials). On the basis of this result, we concluded that the planar lipid bilayer remains stable during incubation under our experimental condition.

### 3.2. Fusion Properties of LUVs with Lipid Droplets

To explore the fusion properties of the LUVs and lipid droplets, we changed the incubation times and phospholipid charges of the LUVs and lipid droplets. The fusion properties of LUVs with droplets composed of DOPC and DOPC/DOPS (molar ratio 1:1) were investigated. Rhodamine-DOPE containing LUVs composed of DOPC, DOPC/DOPS (molar ratio 1:1), and DOPC/DOTAP (molar ratio 9:1) were added to the lipid droplet in the jetting well. The diameters of DOPC/DOPS LUV and DOPC/DOTAP LUVs were 103.5 nm and 112.5 nm, respectively. After incubation for 10, 20, and 30 min, the rhodamine fluorescence of giant lipid vesicles, which were formed by pulsed jet flow, was observed under a CLSM. The fluorescence intensity of rhodamine on the giant lipid vesicles was measured. The amount of rhodamine on giant lipid vesicles was obtained from the fluorescence intensity of rhodamine using a carburation curve of rhodamine amount (Figure S3, Supplementary Materials: (a) (without DOTAP) and (b) (with DOTAP)) vs. fluorescence intensity in the giant lipid vesicles). 

**Figure 3:**
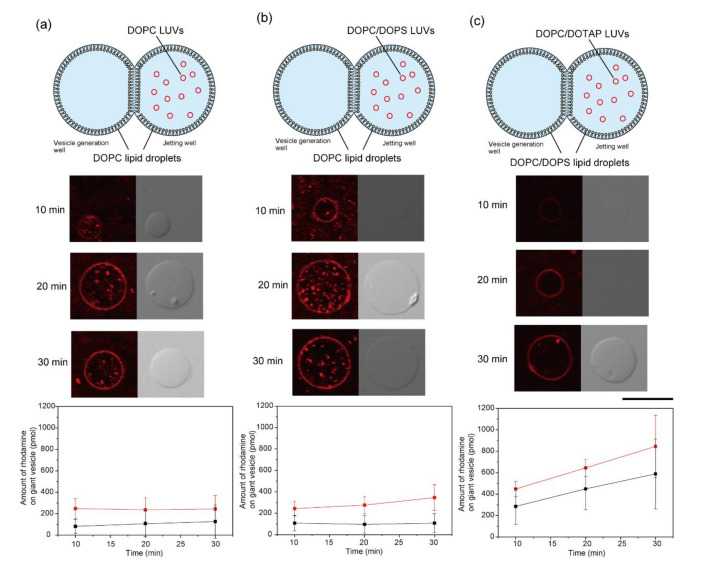
Lipid droplet fusion of rhodamine-DOPE containing LUVs with various lipid compositions. (**a**) DOPC LUVs were fused to DOPC lipid droplets in the jetting well. (**b**) DOPC/1,2-dioleoyl-*sn*-glycero-3-phospho-l-serine (DOPS) LUVs were fused to DOPC lipid droplets in the jetting well. (**c**) DOPC/1,2-dioleoyl-3-trimethylammonium-propane (DOTAP) LUVs were fused to DOPC/DOPS lipid droplets in the jetting well. Typical confocal images of the generated giant lipid vesicles after incubation for 10, 20, and 30 min (CLSM images in (**c**) show low detection). Scale bar, 10 µm. The fluorescence intensity of giant lipid vesicles generated from lipid droplets containing LUVs was measured at various lipid compositions. The red line represents LUV–lipid droplet fusion. The black line represents LUV–giant lipid vesicle fusion as a control experiment. Error bar, standard deviation; *n* > 15 vesicles (over three experiments) at each parameter.

When DOPC LUVs were added to the DOPC droplet, the rhodamine fluorescence intensity on the giant lipid vesicle membrane was not changed following incubation for 10, 20, and 30 min (Figure 3a). The rhodamine fluorescence intensity on the giant lipid vesicle membrane was slightly higher after 30 min of incubation when DOPC/DOPS (molar ratio 1:1) LUVs and DOPC droplets were used compared to that when DOPC LUVs and DOPC droplets were used (Figure 3b). These results indicated that the presence of charged lipids slightly promoted the fusion of LUVs to the lipid monolayer. This slight fusion property is useful for measuring ion currents of a single ion channel in cases where there is an excessively large amount of membrane proteins. Next, we investigated the fusion properties of positively charged LUVs (DOPC/DOTAP, molar ratio 9:1) to a negatively charged lipid droplet (DOPC/DOPS, molar ratio 1:1). The rhodamine fluorescence intensity in this combination was significantly higher than that in the combination of zwitterionic lipid monolayer and LUVs containing zwitterionic lipids or negatively charged lipids (Figure 3c). Therefore, these results suggest that the difference in surface charge between the lipid monolayer and LUVs promoted these fusion phenomena. Next, as a control experiment, LUVs were fused with giant lipid vesicles under the same lipid compositions. The amount of rhodamine on the giant lipid vesicles showed that the fusion of the LUVs to the lipid droplet was greater than that of the LUVs to the giant lipid vesicles (Figure 3a–c). The difference in fusion between the LUVs and the lipid droplet and between the LUVs and the giant lipid vesicle was attributable to the lipid diffusion of the lipid droplet and the giant lipid vesicle; the lipid diffusion of the lipid droplet was higher than that of the giant lipid vesicle [22,23].

### 3.3. Formation of Asymmetric Lipid Vesicles with Rhodamine or GM1

**Figure 4:**
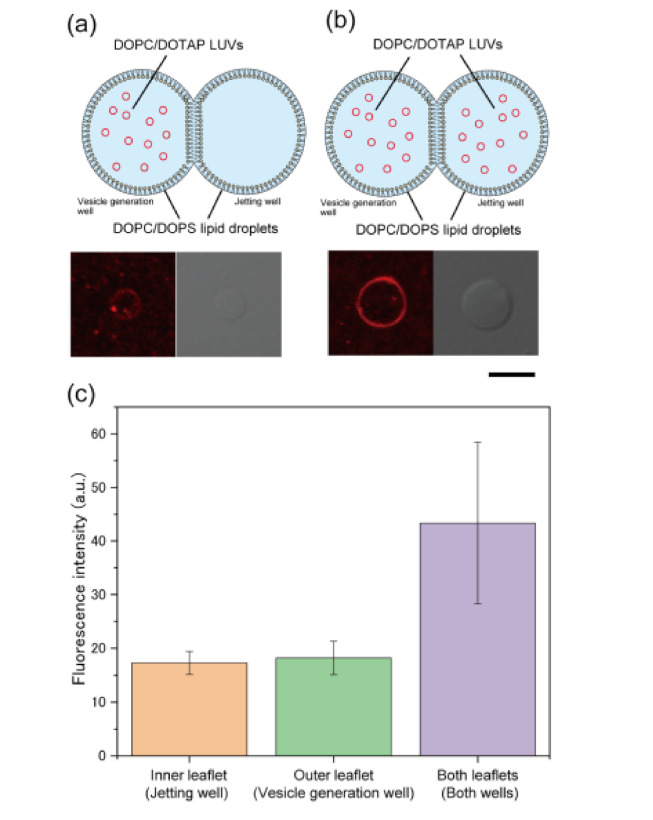
Asymmetric giant lipid vesicle generation via fusion of lipid droplets and LUVs containing rhodamine-DOPE. The figure shows typical confocal images of giant lipid vesicles when DOPC/DOTAP LUVs were fused to DOPC/DOPS lipid droplets in the vesicle generation well only (**a**) or in both wells (**b**). Scale bar, 10 µm. The fluorescence intensity of giant lipid vesicles generated from lipid droplets containing LUVs in the jetting well, vesicle generation well, or both wells was measured (**c**). Error bar, standard deviation; *n* > 10 vesicles (over three experiments) at each parameter.

We investigated whether a symmetric lipid vesicle could be generated using the membrane formed via the fusion of LUVs to a lipid droplet. First, 0.5 mM (final concentration) DOPC/DOTAP (molar ratio 9:1) LUVs were added to DOPC/DOPS (molar ratio 1:1) lipid droplets in the vesicle generation well or both wells (Figure 4a,b). Next, the giant lipid vesicle membrane was formed via pulsed jet flow, and the rhodamine fluorescence intensity on the membrane was measured. The rhodamine fluorescence intensity (mean ± SD) on the giant lipid vesicle membranes formed by the fusion of LUVs to the lipid monolayer in the jetting well and in the vesicle formation well was 17.3 ± 2.1 and 18.2 ± 3.1, respectively (Figure 4c), which was not significantly different. This result indicates that the fusion of LUVs to the lipid monolayer in the jetting well was not promoted by pulsed jet flow. Conversely, when 0.5 mM (final concentration) DOPC/DOTAP LUVs were added to lipid droplets in both wells, the rhodamine fluorescence intensity on the giant lipid vesicle membrane was 43.4 ± 15.3 (Figure 4c), which is more than twice the intensity of the membranes formed from the lipid droplet in the jetting well or the vesicle formation well only. These results suggest that the fusion of LUVs to the lipid monolayer occurred only in the droplet well containing the LUVs. Therefore, this LUV fusion method might enable the formation of giant lipid vesicles with asymmetric lipid distribution (the giant lipid vesicles shown in Figure 2 and Figure 3 might have also had asymmetric lipid distribution). 

**Figure 5:**
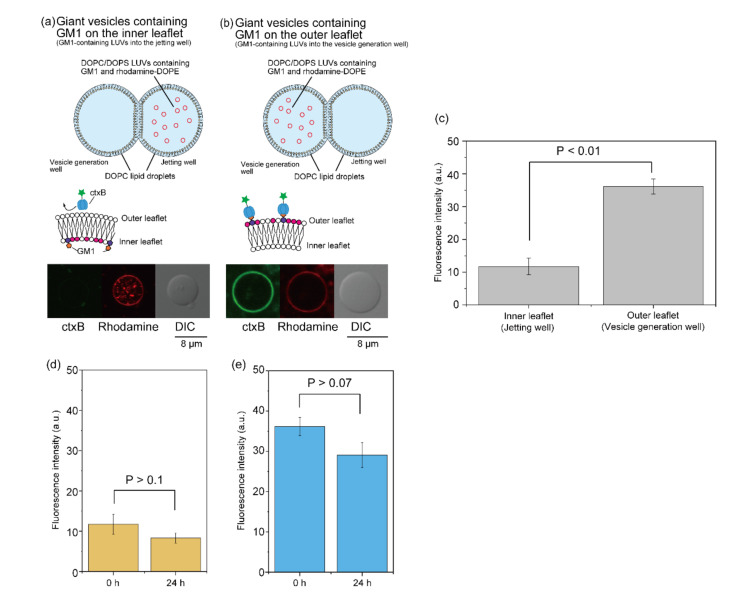
Asymmetric GM1 giant lipid vesicle generation via fusion of lipid droplets and DOPC/DOPS LUVs containing GM1 and rhodamine-DOPE. Typical confocal images of giant lipid vesicles generated from GM1 and rhodamine-DOPE-containing lipid droplets in the jetting well (**a**) or the vesicle generation well (**b**). (**c**) GM1 fluorescence intensity of asymmetric GM1 giant lipid vesicles was measured. Error bar, standard deviation. (**d**) Fluorescence intensity on the asymmetric lipid vesicles with GM1 on the inner leaflet before (*n* = 15 vesicles) or after (*n* = 20 vesicles) incubation for 24 h. Error bar, standard deviation. (**e**) Fluorescence intensity on the asymmetric lipid vesicles with GM1 on the outer leaflet before (*n* = 56 vesicles) or after (*n* = 27 vesicles) incubation for 24 h. Error bar, standard deviation.

To confirm the generation of asymmetric lipid vesicles using this LUV fusion method, 0.5 mM (final concentration) DOPC/DOPS/GM1 LUVs containing rhodamine-DOPE (GM1 LUVs) (DOPC/DOPS/GM1 (molar ratio 4:5:1) containing 76 nM Rh-DOPE) were added to DOPC lipid droplets in the jetting well or the vesicle generation well. The diameter of this LUV containing GM1 was 140.3 nm. After incubation for 20 min at 23 °C, the giant lipid vesicle membrane containing GM1 was generated from the planar lipid bilayer via pulsed jet flow. To estimate the asymmetric distribution of GM1, Alexa Fluor 488-conjugated cholera toxin B subunit (ctxB) was added to the outer solution of the giant lipid vesicle membrane. The detection of Alexa Fluor 488 fluorescence on the giant lipid vesicle membrane proved the existence of GM1 on the outer leaflet. The detection of the rhodamine fluorescence on the giant lipid vesicle membrane proved the fusion of GM1 LUVs to the lipid droplet. When GM1 LUVs were added to the lipid droplet in the jetting well, fluorescence was not observed on the giant lipid vesicle membrane (Figure 5a). On the contrary, when GM1 LUVs were added to the lipid droplet in the vesicle generation well, fluorescence was observed on the cell-sized lipid vesicle membrane (Figure 5b). The fluorescence intensity (mean ± SD) of the cell-sized lipid vesicle membrane generated from the GM1-containing lipid droplet in the vesicle generation well (the outer leaflet of the giant lipid vesicle membrane) and the jetting well (inner leaflet of the giant lipid vesicle membrane) was 36.2 ± 2.3 and 11.7 ± 2.5, respectively (Figure 5c), which was significantly different. We estimated a ratio of fusion of the GM1-conatining LUVs to the lipid monolayer from binding of Alexa Fluor 488-conjugated ctxB to GM1 (see equations in Supplementary Materials). This fusion ratio of the LUVs to the lipid monolayer was 0.073%. The fusion properties of the giant lipid vesicle–LUVs were previously reported. The number of LUVs fused to the giant lipid vesicles was approximately 200 LUVs per 100 µm^2^ of giant lipid vesicle surface [24]. In the case of our method, the number of LUVs fused to the lipid monolayer was approximately 180 LUVs per 100 µm^2^ of giant lipid vesicle surface. This result also indicates that the fusion of the LUVs–lipid monolayer corresponds to the fusion of the LUVs–giant lipid vesicle. The molar ratio of GM1 containing the asymmetric lipid vesicles having GM1 on the outer leaflet was 0.04, corresponding to the molar ratio of GM1 on living cells (0.02) [25]. Therefore, the asymmetric GM1 vesicles formed by our fusion method emulate the lipid compositions of the living cells.

Next, the flip-flop dynamics of GM1 were observed using asymmetric vesicles with GM1 on the outer or inner leaflet. Alexa Fluor 488-conjugated ctxB was added to the outer solution of the GM1 vesicles after incubation for 0 h and 24 h at 37 °C. We observed Alexa Fluor 488 fluorescence on the asymmetric GM1 vesicles before or after incubation for 24 h at 37 °C. The fluorescence intensity (mean ± SD) on the asymmetric lipid vesicles with GM1 on the inner leaflet before and after incubation for 24 h was 11.7 ± 2.5 and 8.3 ± 1.3, respectively (Figure 5d). On the contrary, the fluorescence intensity (mean ± SD) on the asymmetric lipid vesicles with GM1 on the outer leaflet before and after incubation for 24 h was 36.2 ± 2.3 and 29.1 ± 3.1, respectively (Figure 5e). The fluorescence intensities on the asymmetric GM1 vesicles before and after incubation were not significant different. Therefore, we found that GM1 was not transferred through the lipid bilayer membrane in this time scale. These results suggest that lipid vesicles with asymmetric lipid distribution could be generated from the planar lipid bilayer which was formed at the interface between lipid droplets containing and not containing LUVs. To prevent the mixing of lipids via the lipid bilayer during the fusion of LUVs and the lipid droplet, the two lipid droplets were separated by a thin acrylic film [26]. However, our results also showed that the mixing of lipids via the lipid bilayer without the thin acrylic film did not occur during the fusion of LUVs and the lipid droplet. 

The formation of asymmetric lipid bilayers using the BLM system was previously reported. The asymmetric lipid bilayers were formed from two aqueous droplets containing LUVs of different compositions in an organic solvent [27]. Control of the droplet contact area (formation area of the asymmetric lipid bilayer) is difficult due to the small volume of the droplets (approximately 100 nL). Asymmetric lipid bilayers were also formed by contacting two lipid droplets with different lipid compositions into the double-well chamber divided into a separator with a micropore [17]. The use of lipids with poor solubility in *n*-decane is difficult with this asymmetric lipid bilayer formation method. This droplet fusion method enabled control of the formation area of the asymmetric lipid bilayer and formation of asymmetric lipid vesicles containing lipids with poor solubility in *n*-decane. 

## 4. Conclusions

We elucidated the fusion properties between LUVs and lipid droplets by measuring the fluorescence intensity of rhodamine-conjugated lipids on the giant lipid vesicle membrane generated via pulsed jet flow against the planar lipid bilayer, which was formed at the interface between the two lipid droplets. The complementary charge of the LUVs (containing cationic lipids) and lipid droplets (containing anionic lipids) promoted lipid membrane fusion. In every composition of LUVs and lipid droplets, the frequencies of the fusion between the lipid droplet and LUVs were higher than those of the fusion between the giant lipid vesicle and LUVs. When LUVs were added to the lipid droplet in one side of the well, cell-sized asymmetric lipid vesicles were generated from the planar lipid bilayer formed at the interface between the lipid droplets with/without LUVs. This fusion properties between LUVs and the lipid droplet can be applied to measure the efficiency of ion channels on the basis of the artificial planar lipid bilayer fused with the LUVs containing the ion channels. The LUV fusion method will be useful to form lipid vesicles containing lipids with poor solubility in *n*-decane, containing various types of membrane proteins. Therefore, this LUV fusion approach will provide a way for the efficient reconstitution and detection of ion channels on a planar lipid bilayer, as well as the formation of asymmetric lipid vesicles and membrane protein-reconstituted lipid vesicles to create biosensors and artificial cell models [28].

## Supplementary Materials

The following are available online at https://www.mdpi.com/2072-666X/12/2/133/s1: Figure S1: Double-well device setup for generating the giant lipid vesicles; Figure S2: Droplet leakage assay of LUVs; Figure S3: Correlation between fluorescence intensity and mol of rhodamine-DOPE; Figure S4: Correlation between fluorescence intensity and mol ratio of GM1.
