# Supplementary material for: Investigation of Fusion between Nanosized Lipid Vesicles and a Lipid Monolayer Toward Formation of Giant Lipid Vesicles with Various Kinds of Biomolecules
Source: Micromachines (Basel). 2021 Jan 26;12(2):133. doi: 10.3390/mi12020133 (PMC7911008; doi:10.3390/mi12020133)
Supplement: Supplementary file 1 [file micromachines-12-00133-s001.pdf]

Supplementary Materials

Investigation of Fusion between Nanosized Lipid Vesicles and a Lipid Monolayer toward Formation of Giant Lipid Vesicles with Various Kinds of Biomolecules

Koki Kamiya [1,*], Chika Arisaka [1] and Masato Suzuki [2]

[1] Division of Molecular Science, Graduate School of Science and Technology, Gunma University 1-5-1 Tenjin-cho, Kiryu, Gunma 376-8515, Japan; t201a006@gunma-u.ac.jp
[2] Department of Chemistry and Biochemistry, Faculty of Science and Technology, Gunma University 1-5-1 Tenjin-cho, Kiryu, Gunma 376-8515, Japan; t170a072@gunma-u.ac.jp
* Correspondence: kamiya@gunma-u.ac.jp; Tel/Fax: +81-277-30-1342

(a)

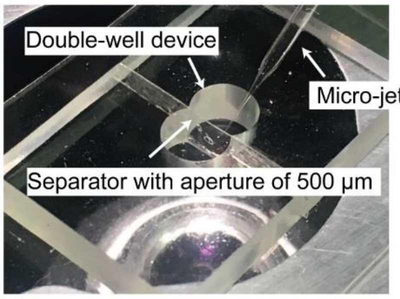

(b)

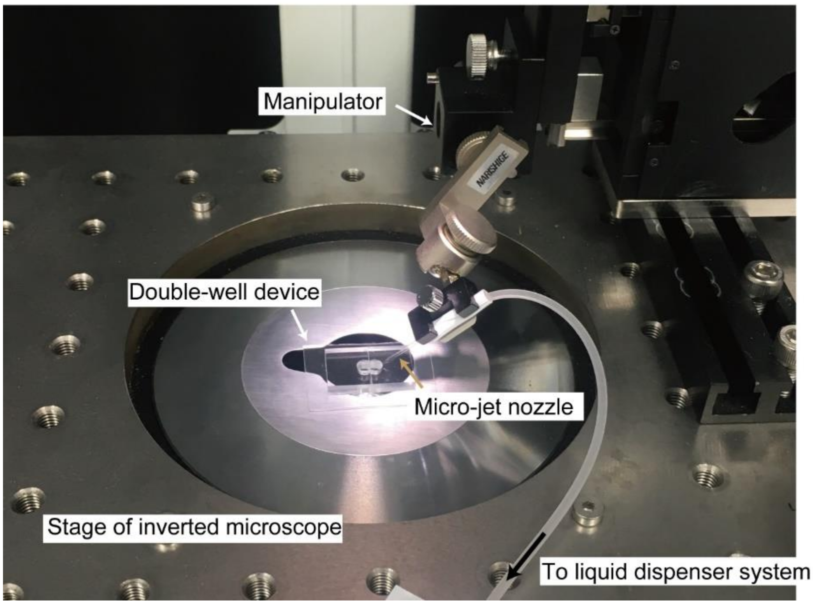

Figure S1. (**a**) Image of double-well device and micro-jet nozzle (there are not phospholipid solution and buffer solution into the double-well device). A planar lipid bilayer is formed into aperture. Micro-jet nozzle is adjacent to planar lipid bilayer by an inverted microscope observation. (**b**) Image of setup of double-well device and micro-jet nozzle on the stage of the inverted microscope. Micro-jet nozzle is connected to a liquid dispenser system.

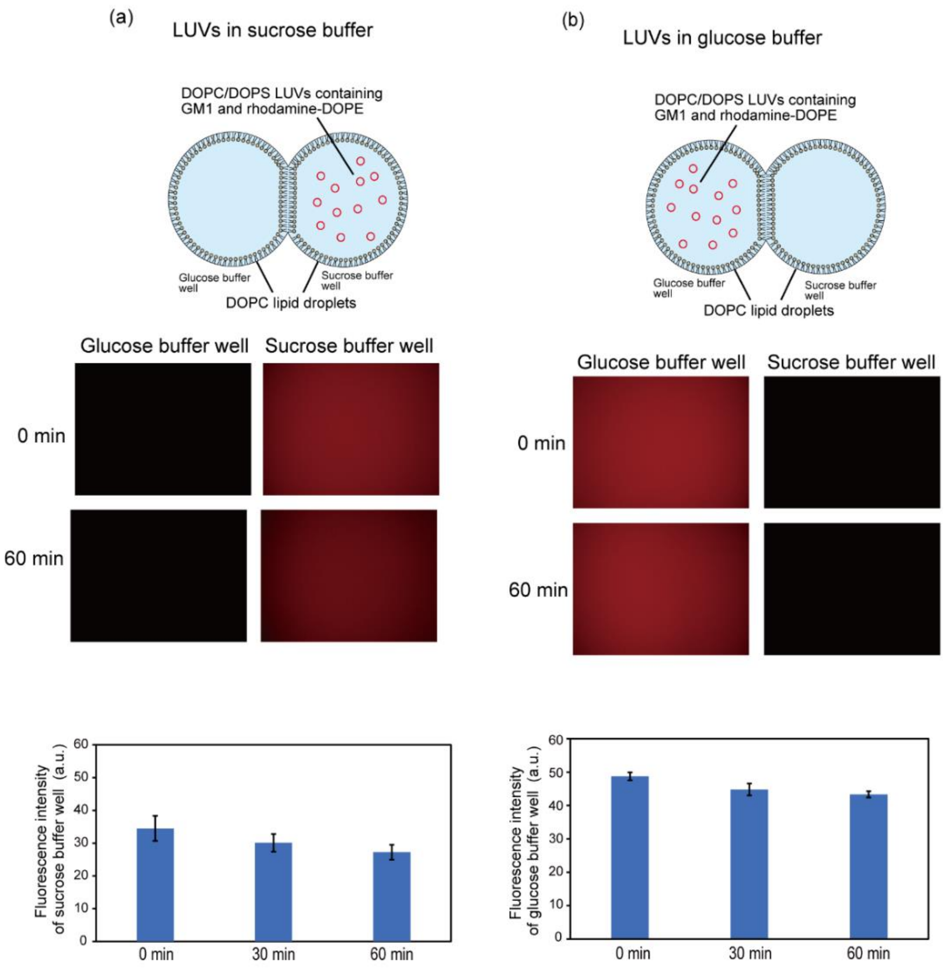

Figure S2. A final concentration of 0.5 mM DOPC LUVs containing rhodamine-DOPE were added to the DOPC droplet in the sucrose buffer (**a**) and the glucose (**b**). The images of the rhodamine fluorescence in the well were acquired using an objective lens (10×) and CCD camera.

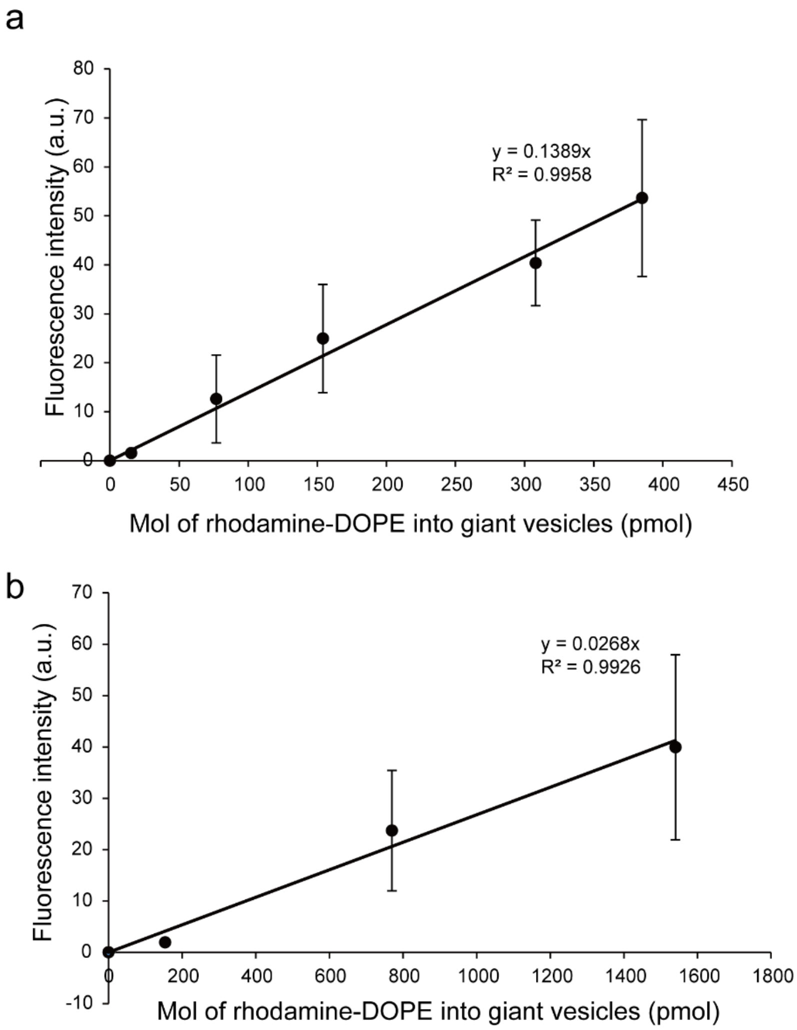

Figure S3. Calibration curve for conversion from fluorescence intensity to mol of rhodamine-DOPE into giant vesicles (pmol). These calibration curves were used for implemental results without DOTAP (**a**) and with DOTAP (**b**).

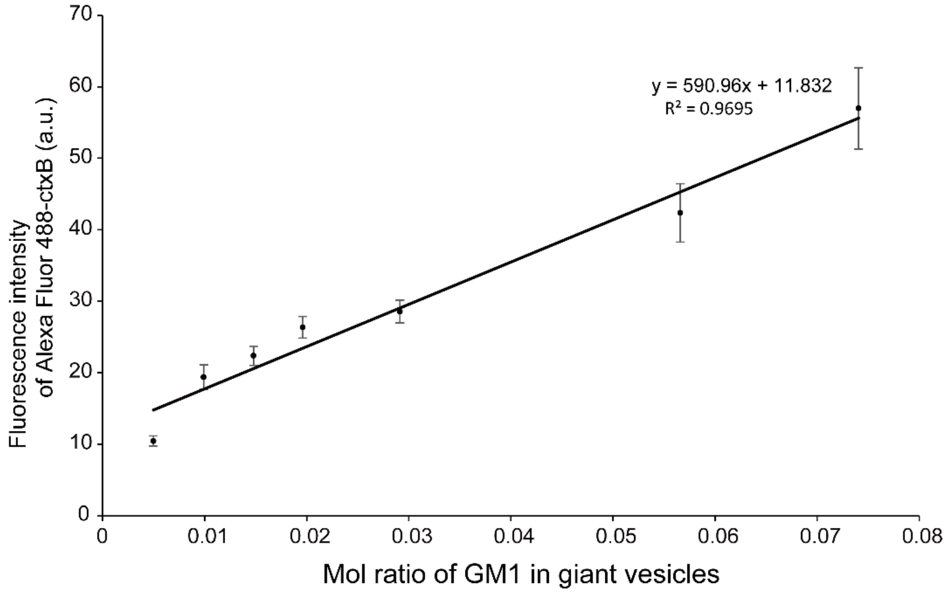

Figure S4. Calibration curve for conversion from fluorescence intensity of Alexa Flour 488-ctxB to molar ratio of GM1 into giant vesicles.

Estimation of fusion ratio of GM1-LUVs to the lipid droplet

The molar ratio of GM1/all lipids in the giant vesicles was estimated from the average fluorescence intensity of Alexa Flour488-ctxB (36.1605) and the calibration curve of Figure S3.

The molar ratio of GM1/all lipids = 0.0412

The number of DOPC on the outer leaflet of the giant vesicles with 10 μm diameter is $4.36x10^8$.

The number of GM1 on the outer leaflet of the giant vesicles is 0.00412 x $4.36x10^8$ = $1.795x10^7$.

The number of DOPC on the both leaflets of the LUVs with 100 nm dimeter is 8.1 $x10^4$.

The molar ratio of GM1 on the both leaflets of the LUVs formed by this experiment is 0.1.

Therefore, the number of GM1 on the both leaflets of the LUVs is 0.1 x 8.1 $x10^4$ = $8.1x10^3$.

The number of fusions of the LUVs to the giant vesicles with 10 μm diameter is

(number of GM1 on the outer leaflet of the giant vesicles)/(number of GM1 on the both leaflets of the LUVs) = ($1.795x10^7$)/($8.1x10^3$) = $2.216x10^3$.

When 23 uL of buffer solution was added to a well of the cylinder shape with 4 mm diameter, the surface area of the lipid monolayer into the well is 35.56 mm^2.

The surface area of the giant vesicles with 10 μm diameter is 1.26 $x10^{-3}$ mm^2.

(The surface area of the lipid monolayer into the well)/(the surface area of the giant vesicles with 10 μm diameter) = $2.831x10^4$.

The number of fusion of the LUVs to the lipid droplet into the well is $2.216x10^3$ x $2.831x10^4$ = $6.27x10^7$.

The number of the LUVs with 100 μm in diameter into the lipid droplet is $8.55x10^{10}$.

Therefore, fusion ratio of the LUVs to the lipid droplet is (The number of fusion of LUVs to the lipid droplet into the well)/(The number of the LUVs into the lipid droplet) = ($6.27x10^7$)/($8.55x10^{10}$) = 0.000734 = 0.073%.
